# Infantile or hypoplastic uterus? A proposal for a modification to the ESHRE/ESGE classification of female genital tract congenital abnormalities

**DOI:** 10.52054/FVVO.14.1.004

**Published:** 2022-04-03

**Authors:** T Küçük, B Ata

**Affiliations:** Acıbadem Maslak Hospital, Dept. of Obstetrics & Gynecology, Istanbul, Turkey; Koç University, School of Medicine, Dept. of Obstetrics & Gynecology, Istanbul, Turkey

**Keywords:** Mullerian anomaly, Hypoplastic uterus, Infantil uterus, T shaped uterus, Y shaped uterus

## Abstract

We argue that the graphical depiction of “infantile uterus” in the ESHRE/ESGE classification of Mullerian anomalies does not fall under class U1b, i.e. uterine corpus anomalies with a normal external contour. The verbal description of “infantile uterus” by the ESHRE/ESGE classification seems to better suit a hypoplastic uterus and as such, arguably, can be omitted from this classification. We also suggest the inclusion of a “Y shaped” uterus under Class U1.

## Introduction

An accurate classification of Müllerian anomalies aims to guide clinicians to a correct diagnosis and appropriate management. We suggest a modification to the ESHRE/ESGE classification of female genital tract congenital abnormalities to improve its accuracy regarding the illustration and classification of “uterus infantilis”, and the inclusion of “Y” shaped uterus (Grimbizis et al., 2013).

## Opinion

**Figure 1:**
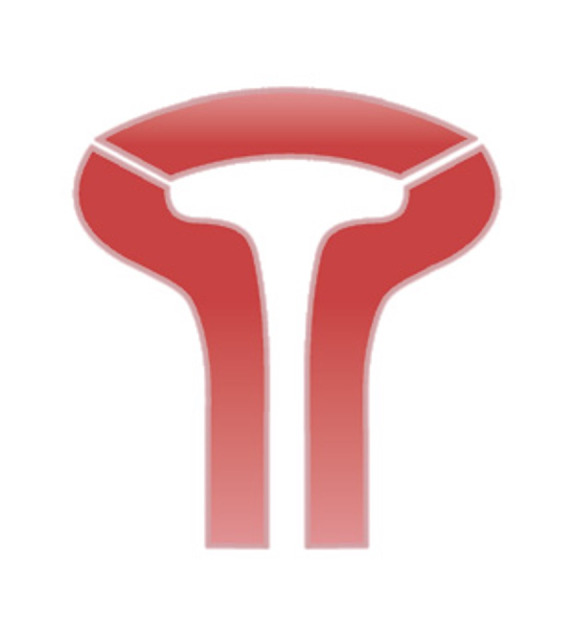
Depiction of uterus infantilis by ESHRE/ESGE classification.

The ESHRE/ESGE classification of female genital tract congenital abnormalities defines Class U1 uterine abnormalities as “All cases with normal uterine outline but with an abnormal shape of the uterine cavity excluding septa”. However, Class U1 b, named “uterus infantilis”, is depicted with a T shaped cavity enclosed with an abnormal uterine outline (Figure 1). This contradicts with the definition of Class U1 which requires a normal uterine outline. Moreover, it is named as “Uterus infantilis” which implies that a normal uterus acquires a T shaped external contour (in addition to a T shaped cavity) during a period of development before reaching its normal adult anatomy. However, we were unable to find any proof of such an “infantil” stage with a T shaped outline or T shaped cavity as graphically depicted by the authors. Yet, the authors’ description of “uterus infantilis” as “…characterized also by a narrow uterine cavity without lateral wall thickening and an inverse correlation of 1/3 uterine body and 2/3 cervix” in the text is correct and consistent with literature (Rauthe and Vahrson 1975). The “infantil uterus” as verbally described by the authors has a normal external contour, characterized with a corpus that has not completed its development and has a normal endometrial cavity with a smaller than adult interostial distance. This conformation can be regarded normal before puberty. However, since the classification is intended for reproductive aged women, a uterus with a normal external contour, short intercornual distance and short length would be named as a “hypoplastic uterus” rather than an “infantil uterus” and would be regarded as abnormal at reproductive age, but perhaps not a congenital anomaly. Arguably, a hypoplastic uterus would not qualify to be included in the ESHRE/ ESGE classification of Müllerian anomalies.

## Conclusion

**Figure 2:**
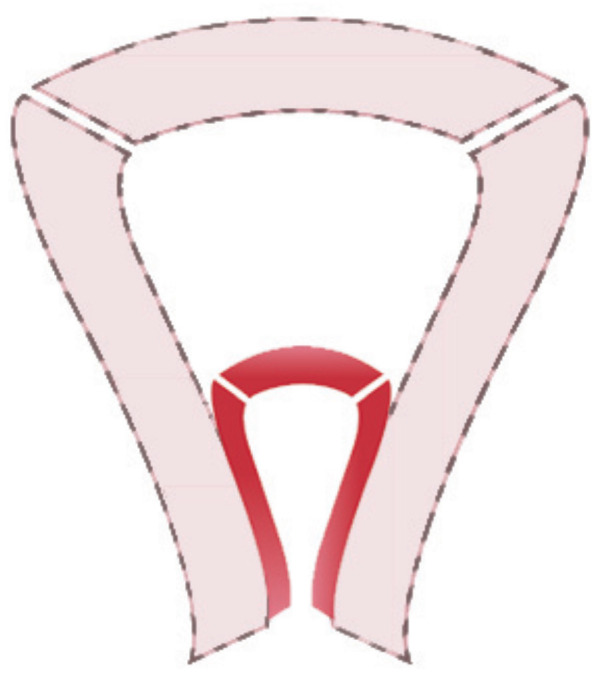
Hypoplastic uterus depicted within a normal sized uterus to give an impression of its smaller than normal size.

We suggest the following modifications. Omission of “infantile uterus” from the text and figures of Class U1. It should be noted that a hypoplastic uterus would have a normal external contour and a normal relationship between the length of the cervix and the length of the uterine cavity of approximately 1:2 in a reproductive aged woman”. Graphic description of “hypoplastic uterus” could be uterine corpus with normal shape accompanied by a hypolastic one showing its smaller than normal size as in Figure 2.

Class U6 would be still for unclassified cases to be described in the future. If the presence of a uterus with a T shaped serosal contour accompanied by a T shaped endometrial cavity as depicted in the original ESHRE/ESGE Class U1 b diagram, is documented it can possibly be placed in Class U6.

**Figure 3:**
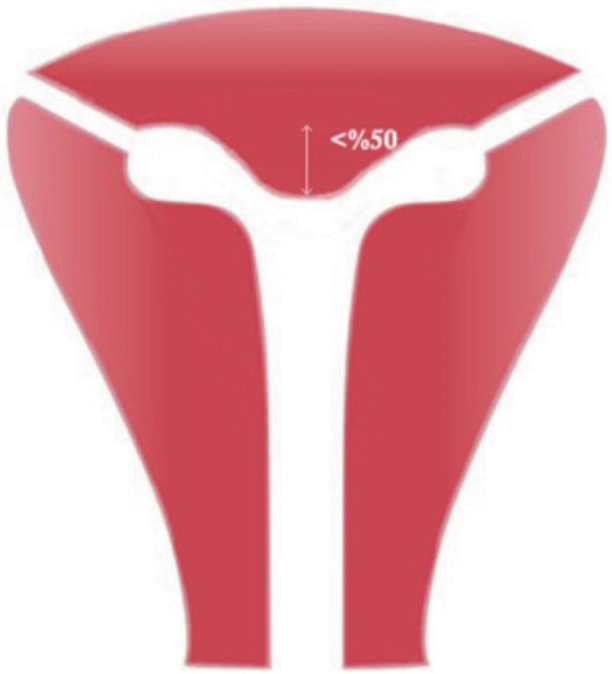
Hypoplastic uterus depicted within a normal sized uterus to give an impression of its smaller than normal size.

We also suggest the addition of a “Y shaped uterus” instead of “uterus infantilis” under U1b (Grimbizis and Campo 2010). Y-shaped uterus could be described as “A uterus characterized by a narrow uterine cavity due to thickened lateral walls and a fundal indentation of <50% of the uterine wall thickness at the midline level, with a correlation 2/3 uterine corpus and 1/3 cervix”. This aims to facilitate differentiating from patients with septate uterus. The Y shaped uterus can be graphically depicted as in the Figure 3.

We think the suggested modifications can improve the accuracy of this comprehensive and easy to use classification by ESHRE and ESGE.
